# Evidence for a Negative Correlation between Human Reactive Enamine-Imine Intermediate Deaminase A (RIDA) Activity and Cell Proliferation Rate: Role of Lysine Succinylation of RIDA

**DOI:** 10.3390/ijms22083804

**Published:** 2021-04-07

**Authors:** Luisa Siculella, Laura Giannotti, Benedetta Di Chiara Stanca, Matteo Calcagnile, Alessio Rochira, Eleonora Stanca, Pietro Alifano, Fabrizio Damiano

**Affiliations:** 1 Laboratory of Molecular Biology, Department of Biological and Environmental Sciences and Technologies, University of Salento, 73100 Lecce, Italy; laura.giannotti@unisalento.it (L.G.); benedetta.dichiara@unisalento.it (B.D.C.S.); alessio.rochira@unisalento.it (A.R.); eleonora.stanca@unisalento.it (E.S.); 2 Laboratory of Microbiology, Department of Biological and Environmental Sciences and Technologies, University of Salento, 73100 Lecce, Italy; matteo.calcagnile@unisalento.it (M.C.); pietro.alifano@unisalento.it (P.A.)

**Keywords:** lysine succinylation, L-PSP, YigF/YER057c/UK114, reactive imine/enamine intermediate deaminase A, 3D protein modeling, metabolic sensor, cancer proliferation

## Abstract

Reactive intermediate deaminase (Rid) proteins are enzymes conserved in all domains of life. UK114, a mammalian member of RidA subfamily, has been firstly identified as a component of liver perchloric acid-soluble proteins (L-PSP). Although still poorly defined, several functions have been attributed to the mammalian protein UK114/RIDA, including the reactive intermediate deamination activity. The expression of UK114/RIDA has been observed in some tumors, arousing interest in this protein as an evaluable tumor marker. However, other studies reported a negative correlation between UK114/RIDA expression, tumor differentiation degree and cell proliferation. This work addressed the question of UK114/RIDA expression in human non-tumor HEK293 cell lines and in some human tumor cell lines. Here we reported that human RIDA (hRIDA) was expressed in all the analyzed cell line and subjected to lysine (K-)succinylation. In HEK293, hRIDA K-succinylation was negatively correlated to the cell proliferation rate and was under the control of SIRT5. Moreover, K-succinylation clearly altered hRIDA quantification by immunoblotting, explaining, at least in part, some discrepancies about RIDA expression reported in previous studies. We found that hRIDA was able to deaminate reactive enamine-imine intermediates and that K-succinylation drastically reduced deaminase activity. As predicted by in silico analysis, the observed reduction of deaminase activity has been related to the drastic alterations of hRIDA structure inferred by K-succinylation. The role of hRIDA and the importance of its K-succinylation in cell metabolism, especially in cancer biology, have been discussed.

## 1. Introduction

Reactive imine/enamine intermediate deaminase A (RIDA) is a member of a large family of YigF/YER057c/UK114 proteins widely distributed in all kingdoms of life. RIDA is involved in removing toxic metabolic intermediates such as 2-aminoacrylate (2AA), able to cause damages to cellular components [1]. 2AA has been firstly identified in *Salmonella enterica* and is generated by pyridoxal phosphate (PLP)-dependent serine/threonine dehydratase and cysteine desulfhydrase [1,2].

2AA forms enamine/imine tautomers. In the imine form, 2AA reacts with PLP cofactor forming a covalent adduct, thus causing the inactivation of PLP-dependent enzymes [1,2]. Beside 2AA, RIDA catalyzes the hydrolysis of other reactive imines formed by L-amino acid oxidase (LAAO) [3,4,5]. Other functions have been attributed to RIDA, such as endoribonuclease activity, inhibitor of protein synthesis, activator of μ-calpain protease, even though they need to be confirmed [6]. Several crystal structures of RIDA proteins have been determined long before the discovery of their enzymatic activity. RIDA proteins are homotrimers with a cleft harboring the active site between two adjacent monomers [5].

UK114, a mammalian member of YigF/YER057c/UK114 proteins, is also known as liver-perchloric acid soluble protein (L-PSP), being this protein detectable in the perchloric acid-soluble protein extract from goat liver [7]. The human homolog of UK114/RIDA is encoded by *Hrsp12* gene, whose expression is high in hepatocytes and in renal distal tubular epithelial cell and low in all the other tissues [8,9].

Contrasting results concern the intracellular localization of RIDA in eukaryotes. Rat and human RIDA has been localized in cytoplasm, peroxisomal matrix, in nucleus, and in mitochondria [8,9,10,11].

Human RIDA (hRIDA) has been described as a tumor antigen in various tumors [12,13]. However, experimental data on the tumor antigenic nature of hRIDA and on its expression are quite controversial. hRIDA was shown to be down regulated in most of hepatocellular carcinoma tissues compared with the adjacent non-tumor hepatic tissue [14]. hRIDA expression was negatively correlated with the tumor differentiation degree [14]. However, overexpression of exogenous RIDA did not suppress the proliferation and tumorigenicity of human hepatoma cells in nude mice [14]. Similarly, hRIDA is expressed in differentiated renal tubules [15], whereas a low expression has been found in kidney tumor cells [8]. On the contrary, data available in The Human Protein Atlas program indicate that RIDA is well expressed in liver and in kidney cancers [16].

In cultured cell lines RIDA expression has been also correlated to cell proliferation, as it was lower in the proliferation phase than in the confluent phase [17]. Moreover, it was reduced in rat after partial hepatectomy and then gradually increased during liver regeneration, achieving the level of the pre-operated liver, after 7 days from partial hepatectomy [18].

Uniprot data (uniprot.org) report that RIDA is subjected to extensive post-translational modifications (PTM), i.e., succinylation of different lysine residues (K-succinylation).

Based on all the observations reported so far and considering the small dimension of the protein (14.5 KDa), we hypothesized that the discrepancies of RIDA expression among the various experimental contexts could be explained by the different sensitivity/specificity of the antibodies used in these studies.

The aim of this study was to deepen the expression of hRIDA in human non-tumor HEK293 cells and in tumor cell lines. Here, we provided the evidence that hRIDA was expressed in several human cell lines. In HEK293, hRIDA underwent K-succinylation, as clearly supported by Western blotting experiments. We also showed that K-succinylation of hRIDA was under the control of Sirtuin 5 (SIRT5). Moreover, the level of hRIDA K-succinylation was not constant during the cell growth phases. In HEK293, hRIDA K-succinylation was low in actively proliferating cells, whereas it was remarkable in low proliferating cells. Now importantly, hRIDA quantification by Western blotting experiments was affected by K-succinylation. Moreover, we found that K-succinylation of hRIDA drastically affected enzymatic activity of either purified recombinant or V5-tagged hRIDA isolated from HEK293. The reduced enzymatic activity of K-succinylated hRIDA might be ascribed to relevant changes in protein structure inferred by the addition of succinyl group on lysine residues, as predicted by in silico analysis. The significance of hRIDA K-succinylation and its correlation with cell proliferation rate has been discussed.

## 2. Results

### 2.1. Analysis of hRIDA Expression in Different Human Cell Lines and Post-Translational Modification

**Figure 1:**
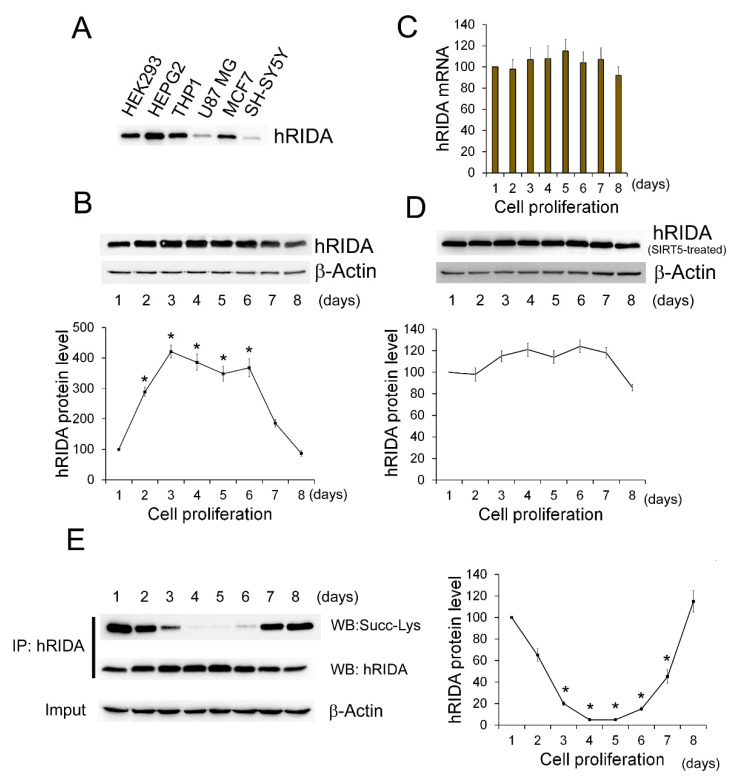
Expression of hRIDA in cultured cell lines: evidence for hRIDA K-succinylation. (**A**) The expression of hRIDA was analyzed in HEK293, HepG2, THP1, U87MG, MCF7 and SH-SY5Y cells by Western blotting. 20 µg of protein extract from each cell line was fractionated on 15% SDS-PAGE, transferred onto nitrocellulose membrane and immunodecorated with the hRIDA polyclonal antibody. The equivalent amount of protein extract from the analyzed cell lines was checked by Ponceau S staining of membrane (data not shown). (**B**) The expression of hRIDA was monitored during HEK293 cell growth, by harvesting cells at several days (1–8 days) following seeding. hRIDA level was determined as described in (**A**) and quantified by densitometry. The content of hRIDA was normalized with β-actin and expressed as percentage with respect to hRIDA content in cells at day 1. (**C**) Cells were harvested at different days of cell growth and total RNA was extracted. hRIDA mRNA abundance was determined by RT-qPCR and normalized with 18S rRNA. Values are reported in histograms as percentage relative to the cells at day 1. (**D**) The content of hRIDA was analyzed in cells harvested at different days (1–8) and protein extracts were subjected to in vitro desuccinylation with SIRT5. The content of hRIDA was determined as described in (**B**). (**E**) hRIDA was immunoprecipitated with polyclonal antibody, fractionated on 15% SDS-PAGE, and transferred onto nitrocellulose membrane. The membrane was immunodecorated using the pan-succinyl-lysine (upper panel) and hRIDA polyclonal (middle panel) antibodies. The content of K-succinylated hRIDA was normalized with respect to β-actin in the input protein extracts (lower panel) and reported in the graph as percentage with respect to the cells at day 1. Values are means ± S.D. of three independent experiments. *, value significantly different from the control, *p* < 0.05.

The expression of hRIDA has been investigated in different cell lines: HEK293, HepG2, THP1, U87 MG, MCF7, and SH-SY5Y cells. Western blotting experiments with RIDA polyclonal antibody indicated that a 15 KDa band corresponding to hRIDA was found in all cell lines (Figure 1A).

In order to investigate the correlation between hRIDA expression and cell proliferation, the non-tumor HEK293 cells, which show the phenomenon of contact inhibition, were used. HEK293 grew with a low proliferation rate in the Lag phase (24 h–48 h). Cells exhibited a logarithmic proliferation (Log) phase from 72 h to 6 days (d) and then entered in the stationary phase 9d after seeding (Supplementary Figure S1).

The level of hRIDA was higher in the Log proliferation phase than in Lag proliferation and the stationary phases (Figure 1B). RT-qPCR experiments showed no change of mRNA level during cell proliferation (Figure 1C).

Data from UniProt (https://www.uniprot.org/, accessed on 1 October 2020) indicate that hRIDA shows post-translational modifications, i.e., acetylation at the N-terminal serine and N6-succinylation at several lysine residues. Owing to the small size of the protein, we hypothesized that K-succinylation of RIDA could interfere with protein quantification by Western blotting, causing misinterpretation of RIDA expression analysis. To test this hypothesis, we quantified the hRIDA level after removing the succinyl residue from K-succinylated proteins, which are observed in all the phases of cell growth in HEK293 (data not shown). SIRT5 is a NAD^+^-dependent deacylase that removes the succinyl residue from succinylated proteins [19]. Thus, the protein extracts were first treated with SIRT5 and then used in Western blotting experiments for the quantification of hRIDA. Results showed that the hRIDA level was almost constant at any time of cell growth (Figure 1D).

Then, to confirm whether hRIDA was effectively K-succinylated in HEK293 cells, hRIDA was first immunoprecipitated from the total protein extracts, and then the level of K-succinylated protein was analyzed by using the anti-succinyl-lysine antibody. Western blotting results indicated that the level of K-succinylated hRIDA was high at 48 h of cell growth, reached the lowest level (5–10%) between 3d–6d, and then incremented in the last phase of cell growth (about 120% at 8d) (Figure 1E).

**Figure 2:**
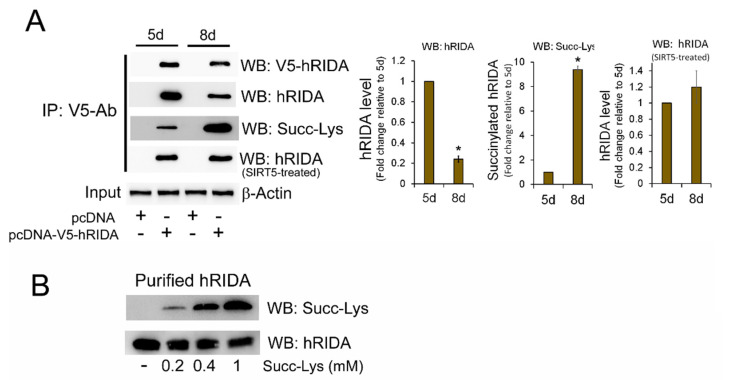
Exogenous V5-tagged hRIDA was differently K-succinylated in high and low proliferating cells. (**A**) High and low proliferating HEK293 cells were transfected with pcDNA-V5-hRIDA or the empty vector pcDNA, at 4d and 7d after seeding the cells. The next day, cells were lysed, and V5-hRIDA was immunoprecipitated from protein extracts using V5 epitope antibody. Immunoprecipitated proteins were fractionated on 15% SDS-PAGE, transferred onto a nitrocellulose membrane and immunodecorated with antibodies against the V5 epitope (WB: V5-hRIDA), hRIDA (WB: hRIDA) and succinyl-lysine (WB: Succ-Lys). V5-hRIDA was in vitro desuccinylated with SIRT5, then transferred onto a nitrocellulose membrane and immunodecorated with the antibody against hRIDA (WB: hRIDA, SIRT5-treated). Chemiluminescence signals were normalized with respect to the β-actin in input protein extracts. Normalized values were reported in the histograms as fold change with respect to the cells at day 5. (**B**) Recombinant purified hRIDA was in vitro succinylated with different concentration of succinyl-CoA, fractionated on SDS-PAGE, transferred onto a nitrocellulose membrane and immunodecorated with antibodies against succinyl-lysine (**B**, upper panel) and hRIDA (**B**, lower panel). Values are means ± S.D. of three independent experiments. *, value significantly different from the control, *p* < 0.05.

A construct carrying V5 epitope-tagged hRIDA cDNA was transfected in HEK293 cells at 4d and 7d after seeding the cells. The empty plasmid pcDNA was used as a control. The level of V5-tagged hRIDA was analyzed at the high proliferation phase (5d) and at the stationary phase (8d) of cellular growth in both pcDNA-V5-hRIDA- and pcDNA-transfected cells. V5-hRIDA content was similar in pcDNA-V5-RIDA-transfected cells at both phases, whereas no signal was detected in control cells transfected with pcDNA (Figure 2A, WB: V5-hRIDA). K-succinylation of immunoprecipitated V5-hRIDA was higher at 8d than that observed at 5d (Figure 2A, WB: Succy-Lys), whereas the hRIDA level was apparently reduced at 8d with respect to 5d (Figure 2A, WB: hRIDA). This apparent difference was not observed when the protein extract was firstly desuccinylated with SIRT5 (Figure 2A, WB: hRIDA, SIRT5-treated). These results showed that hRIDA was K-succinylated in HEK293 cells and the K-succinylation level was dependent on the proliferation phase of cell growth.

In vitro succinylation of lysine residues can be obtained by treating purified proteins with succinyl-CoA [20]. Recombinant purified hRIDA, completely desuccinylated in vitro by SIRT5, was succinylated through the treatment with increasing amount of succinyl-CoA and analyzed by Western blotting. Results showed an increase of K-succinylated hRIDA depending on the succinyl-CoA concentration (Figure 2B, upper panel). Conversely, a reduced signal was observed when hRIDA antibody was used (Figure 2B, lower panel).

### 2.2. K-Succinylation of hRIDA Was Under the Control of SIRT5

**Figure 3:**
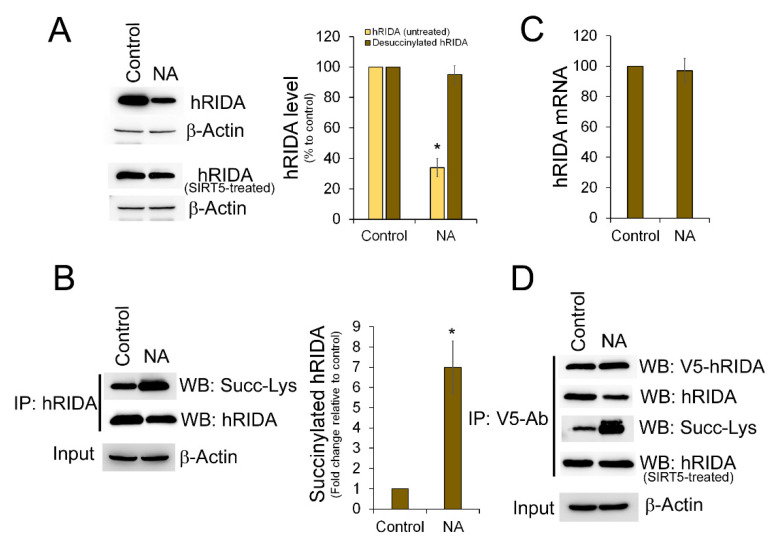
Succinylation of hRIDA was under the control of SIRT5. (**A**) The expression of hRIDA was analyzed by Western blotting in untreated HEK293 cells (control) and in cells treated with nicotinamide (NA) 10 mM for 12 h. 20 µg of untreated (upper panel) and SIRT5-treated (lower panel) protein extract from each sample was fractionated on 15% SDS-PAGE, transferred onto a nitrocellulose membrane and immunodecorated with the hRIDA polyclonal antibody. The content of hRIDA in NA-treated cells was normalized with β-actin and reported as percentage with respect to control cells. (**B**) Endogenous hRIDA was immunoprecipitated from control and NA protein extracts, using hRIDA polyclonal antibody, fractionated on 15% SDS-PAGE, and transferred onto nitrocellulose membrane. The membrane was immunodecorated using the pan-succinyl-lysine (upper panel) and hRIDA polyclonal (middle panel) antibodies. The content of K-succinylated hRIDA was normalized with respect to β-actin in the input protein extracts (lower panel) and reported in histograms as fold change with respect to control cells. (**C**) hRIDA mRNA abundance in untreated (control) and in NA-treated HEK293 cells was determined by RT-qPCR and normalized with 18S rRNA. The amount of hRIDA mRNA in NA-treated cells was reported in histogram as percentage relative to the control. (**D**) V5-hRIDA was immunoprecipitated from control and NA-treated cells as described in Figure 2. Then, proteins were transferred onto a nitrocellulose membrane and immunodecorated with antibodies against V5-epitope (WB: V5-hRIDA), hRIDA (WB: hRIDA) and succinyl-lysine (WB: Succ-Lys). Immunoprecipitated V5-hRIDA was in vitro desuccinylated with SIRT5, and immunodecorated with the antibody against hRIDA (WB: hRIDA, SIRT5-treated). The equivalent amount of protein extract from NA-treated and control cells used in immunoprecipitation was assessed by β-actin analysis in input protein extracts. Values are means ± S.D. of three independent experiments. *, value significantly different from the control, *p* < 0.05.

It has been reported that SIRT5 controls the level of succinylation of several mitochondrial and cytosolic proteins [21]. To evaluate whether SIRT5 regulates the succinylation of hRIDA in vivo, we used nicotinamide (NA) able to inhibit the SIRT5 deacylase activity [22,23]. HEK293 cells were transfected with pcDNA-V5-hRIDA or with the empty plasmid pcDNA, and then treated with NA. Western blotting experiments performed with the anti-RIDA antibody indicated that NA treatment caused a diminution of hRIDA level when compared to the control cells (Figure 3A, upper panel). By contrast, this diminution was not observed when the protein extracts were previously treated with SIRT5 (Figure 3A, lower panel). The K-succinylation of hRIDA was higher in NA-treated cells with respect to control cells (Figure 3B, WB: Succ-Lys). hRIDA mRNA was unaffected upon NA treatment (Figure 3C).

We analyzed K-succinylation of exogenous overexpressed V5-hRIDA in control and in NA-treated cells. Upon immunoprecipitation, V5-tagged hRIDA level was similar in both samples (Figure 3D, WB: V5-hRIDA). An apparent reduction of V5-hRIDA was detected in NA treated cells when the hRIDA polyclonal antibody was used (Figure 3D, WB: hRIDA), whereas the level of K-succinylated V5-hRIDA was higher in NA-treated cells with respect to the untreated cells (Figure 3D, WB: Succ-Lys). Finally, no difference in the hRIDA content was observed when immunoprecipitated samples were pre-treated with SIRT5 (Figure 3D, WB: hRIDA, SIRT5-treated).

**Figure 4:**
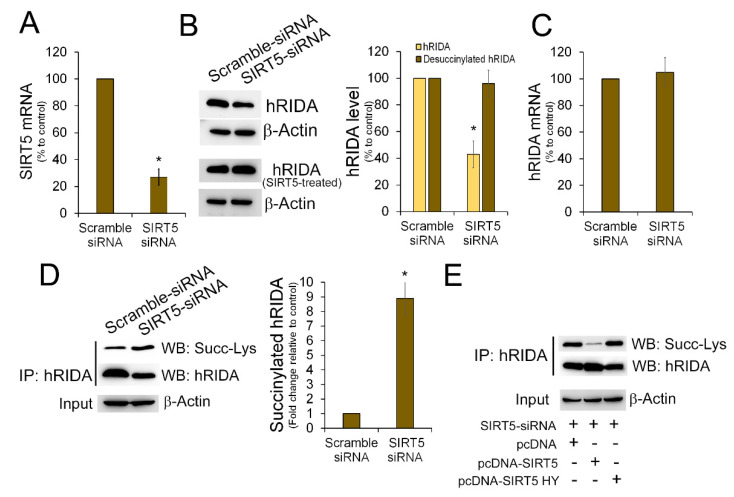
Down-regulation of SIRT5 enhanced hRIDA K-succinylation. (**A**) SIRT5 mRNA amount in scramble siRNA-(control) and in SIRT5 siRNA-transfected cells was determined by RT-qPCR and normalized with 18S rRNA. The abundance of SIRT5 mRNA in SIRT5 siRNA-transfected cells was reported in histogram as percentage of control. (**B**) The expression of hRIDA was analyzed by Western blotting in scramble siRNA-(control) and in SIRT5 siRNA-transfected cells. 20 µg of untreated (upper panel) and SIRT5-treated (lower panel) protein extract from each sample was fractionated on 15% SDS-PAGE, transferred onto a nitrocellulose membrane and immunodecorated with the hRIDA polyclonal antibody. The content of hRIDA in SIRT5 siRNA-transfected cells was normalized with β-actin and reported in histogram as percentage with respect to control cells. (**C**) hRIDA mRNA amount in scramble siRNA-(control) and in SIRT5 siRNA-transfected cells was determined by RT-qPCR and normalized with 18S rRNA. The abundance of hRIDA mRNA in SIRT5 siRNA-transfected cells was reported in histogram as percentage relative to the control. (**D**) hRIDA was immunoprecipitated from scramble siRNA-(control) and in SIRT5 siRNA-transfected cells. Then, proteins were transferred onto a nitrocellulose membrane and immunodecorated with antibodies against succinyl-lysine (upper blot), and hRIDA (middle blot). The content of K-succinylated hRIDA was normalized with respect to β-actin in the input protein extracts (lower blot) and reported in histogram as fold change with respect to control cells. (**E**) HEK293 cells were transfected with SIRT5 siRNA. After 24 h, cells were transfected with the plasmid expressing SIRT5 (pcDNA-SIRT5), mutant SIRT5 H158Y, or the empty vector pcDNA for 24 h and then harvested. Endogenous hRIDA was immunoprecipitated from protein extract, and hRIDA and K-succinylated hRIDA were determined as described in (**D**). Values are means ± S.D. of three independent experiments. *, value significantly different from the control, *p* < 0.05.

The role of SIRT5 in K-succinylation of RIDA was further deepened by down-regulating SIRT5 expression through RNA interference. After transfection with SIRT5-siRNA, the SIRT5 mRNA was reduced with respect to control cells (Figure 4A). In SIRT5-knock down cells the level of endogenous hRIDA was apparently lower when compared to control cells (Figure 4B, upper panel). No change in the level of hRIDA was observed in SIRT5-knock down cells after in vitro deacylation with SIRT5 (Figure 4B, lower panel). Down-regulation of SIRT5 did not cause any change in the hRIDA mRNA level (Figure 4C). K-succinylation of hRIDA was higher in SIRT5-knock down cells with respect to control cells (Figure 4D). Overexpression of wild type (WT) SIRT5, but not of the mutant SIRT5-H158Y, reduced the K-succinylation of hRIDA in SIRT5-knock down cells (Figure 4E). Overall, these results indicate that SIRT5 plays a role in controlling the K-succinylation status of hRIDA in HEK293.

### 2.3. K-Succinylation Negatively Affects Enzymatic Activity of hRIDA

**Figure 5:**
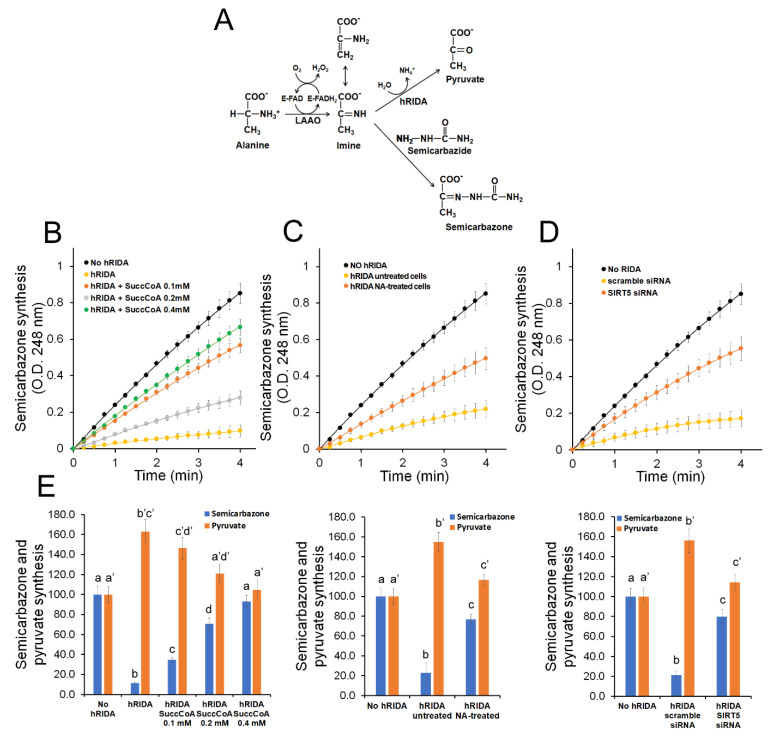
K-succinylation of hRIDA caused a strong reduction of hRIDA deaminase activity. (**A**) Reaction scheme of oxidative deamination of L-amino acids to the corresponding keto-acid catalyzed by the flavoprotein L-Amino Acid Oxidase (LAAO), through the formation of an unstable α-imino acid intermediate. The oxidative deamination of L-alanine generates the toxic compound 2-aminoacrylate (2AA) that tautomerizes to 2-iminopropionate (iminopyruvate), which is hydrolyzed to pyruvate and ammonia, spontaneously or enzymatically by hRIDA. The α-imino acid (iminopyruvate) intermediate reacts with semicarbazide to form the corresponding semicarbazone, which can be determined spectrophotometrically. (**B**) Time course of semicarbazone synthesis reactions carried out in the absence (no hRIDA) or in the presence of unmodified hRIDA (hRIDA) or hRIDA K-succinylated, with increasing concentration of succinyl-CoA (hRIDA + SuccCoA 0.1 mM, 0.2 mM, 0.4 mM). (**C**) Time course of semicarbazone synthesis reactions carried out in the absence (no hRIDA) or in the presence of hRIDA immunoprecipitated from untreated (hRIDA untreated cells) or nicotinamide (hRIDA NA-treated cells) cells. (**D**) Time course of semicarbazone synthesis reactions carried out in the absence (no hRIDA) or in the presence of hRIDA immunoprecipitated from scramble siRNA-transfected (scrambe siRNA) or from SIRT5 siRNA-transfected (SIRT5 siRNA) cells. (**E**) Semicarbazone and pyruvate synthesized in the hRIDA enzymatic assays as described in (**B**–**D**). Values are reported in histograms as percentage relative to the semicarbazone and pyruvate synthesized in the absence of hRIDA (No hRIDA). Values are means ± S.D. of three independent experiments. Within the same group, samples bearing different letters differ significantly (*p* < 0.05).

The effect of K-succinylation on the enzymatic activity of hRIDA was evaluated by a spectrophotometric assay. 2AA was generated by the oxidative deamination of L-alanine catalyzed by snake venom L-amino acid oxidase (LAAO). Then it rapidly reacts with semicarbazide forming the semicarbazone, which can be spectrophotometrically measured (Figure 5A). The activity of hRIDA was evaluated through the reduction of the semicarbazone synthesis rate, due to the prior hydrolysis of the imine by the enzyme (Figure 5A) [5].

A rapid semicarbazone synthesis was observed when LAAO was incubated with L-alanine and semicarbazide, in the absence of hRIDA (Figure 5B). Addition of 0.1 µM recombinant non-succinylated hRIDA caused a strong decrease in semicarbazone formation. We observed that the enzymatic activity of hRIDA was progressively reduced, based on its K-succinylation level (Figure 5B).

Enzymatic activity was also evaluated on V5-hRIDA immunoprecipitated from NA-treated cells and compared with the respective control cells. Results showed that the activity of hRIDA from NA-treated cells was strongly reduced with respect to that obtained from control cells (Figure 5C). Analogously, the deaminase activity of V5-hRIDA immunoprecipitated from SIRT5 knock-down cells was lower with respect to that observed in control cells (Figure 5D).

To confirm that the decrease in semicarbazone formation was due to hRIDA-mediated hydrolysis of imine, we measured both semicarbazone and pyruvate formation. The decrease in semicarbazone formation in the presence of hRIDA was accompanied by an increase in pyruvate formation (Figure 5E). An increment of semicarbazone, accompanied by a reduction in the synthesis of pyruvate, was observed when the reactions occurred in the presence of succinylated hRIDA (Figure 5E).

### 2.4. In Silico Analysis of K-Succinylation Influence on hRIDA Structures

**Figure 6:**
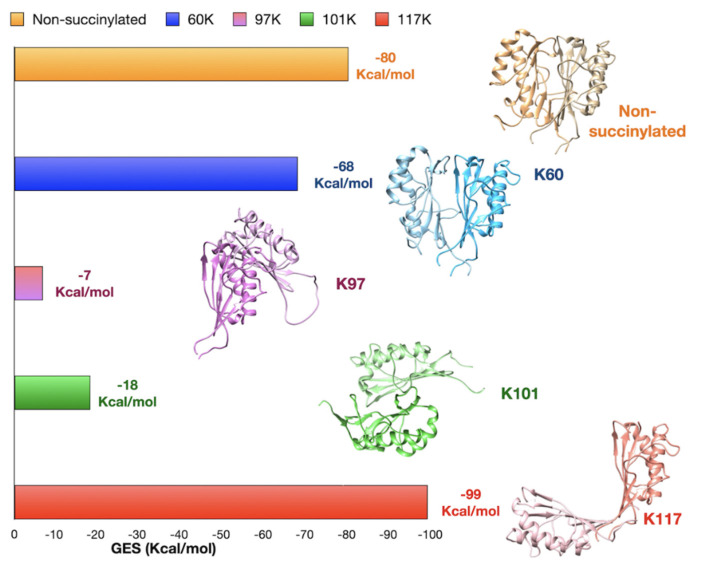
In silico analysis of hRIDA structure changes following K-succinylation. The global energy score (GES, Kcal/mol), calculated by the FireDock program for unsuccinylated hRIDA dimer and K60, K97, K101 or K117 succinylated hRIDA, is reported in histograms. The predicted 3D model of unsuccinylated and K-succinylated hRIDA are reported, highlighting the structural changes inferred by succinyl residue on K60, K97, K101 or K117.

In silico analysis has been performed to evaluate potential alterations of the structure of the hRIDA following K-succinylation. Prediction of hRIDA monomer structure has been obtained with I-Tasser [24] using multiple templates from RCSB PDB database. Based on the predicted model, the hRIDA monomer is folded to form two α-helix and a β-sheet (Supplementary Figure S2A). As suggested by GalaxyHomomer software [25], three hRIDA monomers oligomerize to form a trimeric structure (Supplementary Figure S2B), with the β-sheet and the α-helices directed inwards and outwards, respectively. Several bonds are established between amino acids at the interface of two contiguous monomers, stabilizing the trimer. These amino acids, identified with GalaxyHomomer or CD search, are mainly located on the alpha-helices (Supplementary Figure S2C). Moreover, amino acids in the putative catalytic site have been also identified (Supplementary Figure S2C). Importantly, three lysine residues subjected to succinylation (K97, K101 and K117) are located neighboring to the cluster of amino acids involved in the stabilization of the trimeric structure of hRIDA (Supplementary Figure S2B,C). Based on these predictions, K-succinylation could alter hRIDA trimeric structure. To strengthen this hypothesis, several docking simulations were carried out with native or K-succinylated forms of hRIDA, by using GRAMM-X/FireDock software [26,27]. Docking simulations were performed on hRIDA dimers to obtain the Global Energy Score (GES, Kcal/mol), a parameter that indicates the stability of the complex (Figure 6). These predictions showed that, when compared to native protein, K60, K97 or K101 succinylation drastically altered the structure of hRIDA dimers, thus reducing the complex stability (Figure 6). An antiparallel structure was predicted for K101 succinylated hRIDA complex. The K117 succinylated hRIDA dimer had a low GES value, although it exhibited an open structure (Figure 6).

## 3. Discussion

RIDA is a member of the broadly conserved YjgF/YER057c/UK114 family present in all domains of life [2]. Such broad conservation suggests a critical role of RIDA maintained throughout the evolution. The importance of RIDA has been related to the detoxifying function from reactive enamine/imine intermediates, produced by PLP-dependent threonine/serine dehydratases [2]. Reactive enamine/imine intermediates, previously believed to have a relatively short half-life and rapidly non-enzymatically hydrolyzed into keto acid products, could accumulate in vivo, thus affecting cell metabolism [2]. Although there is currently no evidence on the accumulation of the reactive enamine/imine intermediates in eukaryotic cells, the conservation of RIDA across all domains of life supports the hypothesis that hRIDA plays a detoxifying function also in mammals and in humans. As shown in the present work hRIDA was able to hydrolyze enamine/imine (Figure 5), in agreement with the results reported in other organisms [2,3].

The aim of this study was to investigate the expression of hRIDA in mammalian cells, particularly in human tumor cells. Previous studies identified hRIDA/UK114 as a tumor antigen in various human tumors [12,13]. Recently, it has been reported an opposite behavior. Down-regulation of hRIDA has been observed in liver and kidney tumors [8,9], and has been strictly correlated to the differentiation of tumor cells [14,15]. Another link exists between the expression of RIDA and cell proliferation, a typical feature of cancer cells [17,18]. On the contrary, other data indicated that hRIDA is highly expressed in cancer cells from liver and kidney, as reported in The Human Protein Atlas [16]. Importantly, overexpression of hRIDA in a xenograft model did not suppress the proliferation and tumorigenicity of hepatoma cells, thus ruling out a tumor suppressor function for hRIDA [14].

Besides the evidence of the effective hRIDA expression in various tumor cell lines (Figure 1), an apparent change of the protein level in the cell growth phases has been observed in HEK293 cells (Figure 1). An interesting aspect emerging from this work concerns the succinylation of hRIDA lysine residues. In HEK293 cells, K-succinylated hRIDA level changed in different phases of cell proliferation (Figure 1). hRIDA was faintly K-succinylated in actively proliferating cells, while strong K-succinylation was observed in low proliferating cells, i.e., in the Lag phase and in the stationary phase when cells reached a high level of cellular confluence (Figure 1). Upon in vitro desuccinylation, the differences in the protein levels in the phases of cellular growth were almost nullified. Based on these results, post-translational modification of hRIDA alters the quantification by Western blotting analysis, and this may explain contrasting results about hRIDA expression in previous studies. However, the finding that hRIDA quantification by Western blotting analysis is affected by its succinylation level, requires further confirmation using anti-hRIDA antibodies from different sources.

Desuccinylation of proteins is controlled by SIRT5, a NAD^+^-dependent deacylase able to remove succinyl, malonyl or glutaryl residue from the ε-amino group of the modified lysine in proteins [28,29]. SIRT5 is mainly located in mitochondria, although a cytosolic SIRT5 isoform has been also identified [30]. When compared with untreated control cells, K-succinylation of hRIDA was higher in cells treated with nicotinamide, a SIRT5 inhibitor (Figure 3). This result was further confirmed by SIRT5 down-regulation experiments (Figure 4). Moreover, in SIRT5-knockdown cells, the level of K-succinylation of hRIDA was reduced following over-expression of the WT SIRT5 but not of the mutant SIRT5-H158Y (Figure 4). Therefore, we can conclude that K-succinylation of hRIDA is under the control of SIRT5.

Lysine, an essential amino acid for humans with a positively charged side chain at physiological pH, is fundamental for protein-protein interactions and plays a role in catalytic site of some enzymes. Succinylation neutralizes the positive charge (+1) of lysine, giving it a net negative charge (−1). Accordingly, the shift in the charge by K-succinylation could cause structural alteration with significant effects on the function of hRIDA.

In agreement with previous crystallographic data [31], in silico programs predicted for hRIDA a trimeric structure, stabilized by bonds between amino acids at the interface of two contiguous monomers. Importantly, the cleft between two adjacent monomers harbors the active site [5].

hRIDA contains 8 lysine residues, four of them are succinylated [32]. Of these, three lysine residues (K97, K101 and K117) are located near the interface of two monomers. Not surprisingly, the prediction of the structures of hRIDA with succinyl-residue on K97, K101 and K117 suggested a consistent structure alteration, which could affect the activity of hRIDA (Figure 6).

K-succinylation plays a critical role in regulating various cellular metabolic pathways [28,29]. Succinyloma data revealed that numerous cytosolic, mitochondrial, and nuclear proteins, including metabolic enzymes involved in fatty acid synthesis, fatty acid oxidation, amino acid degradation, mitochondrial respiration, and the tricarboxylic (TCA) acid cycle are K-succinylated. The effect of K-succinylation on the enzyme activity is not always unique. Indeed, it has been observed that K-succinylation positively affects mitochondrial pyruvate dehydrogenase complex (PDC) and succinate dehydrogenase (SDH) activities, thus favoring Krebs cycle and cellular respiration [28]. Conversely, K-succinylation of carbamoyl phosphate synthase 1 (CPS1) [19] and of serine hydroxymethyltransferase 2 (SHMT2), involved in the urea cycle and in folate cycle, respectively, inhibited their enzymatic activity [23].

Our results showed that K-succinylation caused an inhibition of hRIDA deaminase activity, as confirmed by assays performed with the purified recombinant, or exogenous overexpressed V5-hRIDA or endogenous hRIDA. Purified hRIDA activity was progressively inhibited depending on its K-succinylation level (Figure 2 and Figure 5). In the same way, the activity of the endogenous hRIDA or the exogenous overexpressed V5-hRIDA was high in actively proliferating cells (Figure 1 and Figure 5), where the enzyme was almost in the desuccinylated form (Figure 1 and Figure 5). These results suggest that hRIDA must be desuccinylated in rapidly replicating cells, in which there is a more active cell metabolism, especially amino acid metabolism. Future studies on the levels of unmodified/modified lysines and the complex dynamics of hRIDA K-succinylation, using experimental approaches with high performance such as mass spectrometry, could highlight the physiological role of this post-translational modification in the regulation of metabolism and cell proliferation.

It is widely recognized that cancer cells have a particularly pronounced amino acid metabolism [33,34]. Amino acids have a plastic function for the synthesis of new proteins, lipids and nucleotides, which are necessary to support cell proliferation [33,34]. Biosynthesis of glutathione, whose elevated levels are correlated with oxidative stress resistance, tumor growth and metastasis in many cancer cells [35], is also dependent on amino acids metabolism. In all the above-mentioned metabolic pathway, are present PLP-dependent enzymes, whose enzymatic activity might be preserved by hRIDA, in proliferative tumor cells. Therefore, it would be interesting to analyze the succinylation of hRIDA in speciments or cell lines of tumor origin. Overall, the data obtained in this work gain a deeper insight into the possible role of hRIDA in cancer biology.

## 4. Materials and Methods

### 4.1. Cell Culture and Growth Curve Analysis

HEK293, MCF7, HepG2, THP1, U87 MG cell lines were maintained in Dulbecco’s modified Eagle’s medium (DMEM) (Sigma-Aldrich, Milan, Italy) supplemented with 10% (*v*/*v*) heat-inactivated fetal bovine serum (FBS), penicillin G (100 units/mL) and streptomycin (100 μg/mL). SHSY-5Y cells were maintained in DMEM/F12 medium (Sigma-Aldrich, Milan, Italy) with the same supplements as above. Cells were kept at 37 °C in a humidified atmosphere containing 5% CO_2_. For growth curve analysis, 5 × 10^4^ HEK293 cells were seeded in 100 mm dishes and cultured in DMEM with 10% FBS up to 9 days. Medium was refreshed every 48 h. At the indicated times, cells were harvested by trypsinization and counted with a Burker chamber.

### 4.2. Isolation of RNA from Cultured Cells and Real-Time qPCR Analysis

RNA extraction and reverse transcription were carried out as previously reported [36]. Quantitative gene expression analysis was carried out on CFX Connect™ Real-Time PCR Detection System (Bio-Rad Laboratories, Segrate, Italy), using SYBR^®^ Select Master Mix for CFX (Life Technologies) and 18S rRNA for normalization [37]. The primers used for real-time PCR analysis were as follows hRIDAfor (5′-GGAGGGGTAGCAGAAGAAG-3′); hRIDArev (5′-TGAAGTCATTTATGTCAGCCAG-3′); hSIRT5for (5′-GTCCACACGAAACCAGATTTGCC-3′); hSIRT5rev (5′-TCCTCTGAAGGTCGGAACACCA-3′).

### 4.3. Western Blotting Analysis and Immunoprecipitation

Cell protein extract was prepared as previously described [38]. 20 μg of proteins was separated on 15% (*w*/*v*) SDS gels. After electrophoresis, proteins were transferred electrophoretically onto nitrocellulose membrane (Pall, East Hills, NY, USA), that was blocked with 5% (*w*/*v*) non-fat dried milk in buffered saline. Blots were then incubated with specific primary antibodies directed against RIDA (HPA023489, Sigma-Aldrich, Milan, Italy), V5 epitope (V8012, Sigma-Aldrich, Milan, Italy) and β-actin (sc-47778, Santa Cruz Biotechnology, Heidelberg, Germany). The immune complexes were detected using peroxidase-conjugated secondary antibodies by chemiluminescence (ECL WesternBright, Advansta, San Jose, CA, USA). Densitometric analysis was carried out on blots using the NIH Image 1.62 software (National Institutes of Health, Bethesda, MD, USA), normalizing to β-actin used as an internal control.

For immunoprecipitation assay, the culture medium was removed, and cells were washed in cold phosphate-buffered saline (PBS). 1 × 10^7^ cells were lysed in 1 mL cold lysis buffer 50 mM Tris-HCl (pH 7.9), 137 mM NaCl, 1% Triton X-100, 0.2% Sarkosyl, 1 mM NaF, 1 mM Na_3_VO_4_, and 10% glycerol, protease inhibitor cocktail, 1 mM dithiothreitol (DTT), and 1 mM phenylmethyl sulfonyl fluoride (PMSF). Cell debris was removed by centrifuging lysate for 10 min at 8200× *g* at 4 °C. The supernatant was collected and used in the immunoprecipitation and as input for the normalization in Western blotting experiments. The supernatant was incubated with 5 µL of antibody against RIDA or V5-epitope for 1 h 4 °C. Immunoprecipitation were carried out by incubating the lysate with EZview™ Red Protein A Affinity Gel (Sigma-Aldrich, Milan, Italy) for 4 h at 4 °C. After the incubation, beads were washed for four times with ice-cold buffer 20 mM Tris-HCl pH 7.9, 100 mM NaCl, 0.2% NP-40, and 20% glycerol, protease inhibitor cocktail (Sigma-Aldrich, Milan, Italy), 1 mM DTT and 1 mM PMSF (Sigma-Aldrich, Milan, Italy). The beads were then boiled in Laemmli buffer and the supernatants were used to immunoblot with antibody against anti–pan succinyl-lysine (PTM, #419, with 1:1000 dilution). All blots are shown in Supplementary Figure S3.

### 4.4. Plasmids and Recombinant Proteins Synthesis

The expression vector pET-hRIDA was generated by inserting the cDNA fragments from pcDNA DEST40-human L-PSP-V5 (a gift from Dr. H. Kanouchi, Osaka Prefecture University, Japan) between the NdeI and HindIII sites of pET-21b (Novagen-Merck, Milan, Italy) and then used for bacteria transformation.

pET15b-SIRT5 was a gift from Dr. David B. Lombard-University of Michigan [39]. Recombinant hRIDA and SIRT5 were expressed in *E. coli* BL21(DE3), as previously described [40]. After bacteria lysis, recombinant proteins containing C-terminal polyhistidine (His6)-tag was purified to homogeneity by Ni^2+^-Charged resin (Bio-Rad Laboratories, Italy) chromatography. pcDNA-hSIRT5 was a gift from Dr. Yingming Zhao (University of Chicago). The cDNA of mutant hSIRT5HY was obtained from pET15b-SIRT5HY and cloned into pcDNA3.

### 4.5. RNA Interference Analysis

*SIRT5* gene silencing in HEK293 cells was performed by RNA interference with synthetic siRNA (Silencer^®^ Select Pre-designed siRNA, Ambion-Life Technologies; FlexiTube siRNA, Qiagen, Milan, Italy) directed at the sequences in the 3′-UTR region. A siRNA with a scrambled sequence was used as a negative targeting control. Transfection was performed by using HiPerFect Transfection Reagent (Qiagen, Milan, Italy) for 24 h [41] The expression of SIRT5 was rescued by co-transfecting cells with SIRT5-directed siRNA together with pcDNA-SIRT5. Co-transfection using the mutant pcDNA-SIRT5-H158Y was carried out as negative control.

### 4.6. In Vitro hRIDA Desuccinylation and Succinylation Assays

In vitro desuccinylation assay of either purified recombinant hRIDA and HEK293 cell protein extracts, and succinylation of purified recombinant hRIDA were carried out as previously reported [23]. Since protein succinylation is a physiological process occurring in *E. coli* [42], purified recombinant hRIDA was desuccinylated before using it in the succinylation assay. About 30 µg of purified hRIDA was first treated with 2 µg of SIRT5 and 1 mmol/L NAD^+^ in 100 µL desuccinylation buffer at 30 °C for 2 h [23]. Then, desuccinylated hRIDA was newly purified by Ni^2+^-charged resin chromatography and used in K-succinylation assay. In the K-succinylation reaction, hRIDA (100 ng) was incubated in the succinylation buffer in the presence of succinyl-CoA (0.1 mM, 0.2 mM, 0.4 mM) at 30 °C for 15 min [23].

### 4.7. hRIDA Enzymatic Assay

Enzymatic assay was carried out to evaluate the ability of hRIDA to hydrolyze reactive intermediate enamine/imine produced by L-amino acid oxidase (LAAO) as previously reported [5].

The assay was conducted in 100 μL reaction containing 100 mM phosphate buffer, pH 8.0, 10 mM semicarbazide-HCl, bovine liver catalase (24 Units), 0.4 μM L-amino acid oxidase and 0.1 μM of hRIDA. Reaction was started by adding L-alanine to a final concentration of 2 mM, and absorbance at 248 nm was monitored at 22 °C. The molar extinction coefficient for semicarbazone (ε = 10,300 M^−1^ cm^−1^) was used to calculate the rate of semicarbazone synthesis. For pyruvate measurement, after 4 min, the reaction above described was incubated at 90 °C for 10 min to inactivate LAAO. 20 μL aliquot of the stopped reaction was added to assay (100 μL) containing 50 mM Tris-HCl, pH 8.0, 150 μM NADH, 5 units of rabbit muscle lactic dehydrogenase (Sigma-Aldrich, Milan, Italy), and incubated for 10 min at 22 °C while monitoring absorbance at 340 nm. The activity of lactic dehydrogenase was unaffected by 2 mM semicarbazide.

### 4.8. 3D Modeling and Docking Simulations

I-Tasser software [24] was used to construct the hRIDA monomer model, using P52758 (RIDA_HUMAN) Uniprot sequence as query. The PDB file obtained by I-Tasser was used to predict hRIDA trimers by GalaxyHomomer software [25]. Results were visualized by UCSF Chimera [43]. Avogadro software [44] was used to add succinyl group to K60, K97, K101 and K117 in 3d hRIDA model. Dimeric 3D model of unsuccinylated hRIDA or of K60, K97, K101 and K117 succinylated hRIDA was predicted using GRAMM-X [26]. Interaction refinement in molecular docking and the global energy score (GES) evaluation of hRIDA complexes were obtained with FireDock [27]. The analysis of conserved amino acids of hRIDA was carried out with NCBI Conserved Domain Search (CD search, https://www.ncbi.nlm.nih.gov/Structure/cdd/cdd.shtml, accessed on 1 December 2020).

### 4.9. Statistical Analysis

Statistical analysis was performed with SPSS software (IBM SPSS, Inc., Chicago, IL, USA). Data are presented as means ± S.D. and compared using one-way ANOVA and Tukey’s test as post hoc analysis. *p* < 0.05 or less was considered to be statistically significant.

## Data Availability

The data presented in this study are available on request from the corresponding author.

## Supplementary Materials

The following are available online at https://www.mdpi.com/article/10.3390/ijms22083804/s1, Figure S1: HEK cell proliferation curve. HEK293 proliferation curve was obtained by seeding and counting cells in 1–12 days period, Figure S2: In silico analysis of hRIDA 3D trimeric structure, Figure S3: Western blots with marker sizes.
