# Supplementary material for: Evidence for a Negative Correlation between Human Reactive Enamine-Imine Intermediate Deaminase A (RIDA) Activity and Cell Proliferation Rate: Role of Lysine Succinylation of RIDA
Source: Int J Mol Sci. 2021 Apr 7;22(8):3804. doi: 10.3390/ijms22083804 (PMC8067581; doi:10.3390/ijms22083804)
Supplement: Supplementary file 1 [file ijms-22-03804-s001.pdf]

International Journal of
Molecular Sciences

MDPI

Supplementary Materials

Evidence for a Negative Correlation between Human Reactive Enamine-Imine Intermediate Deaminase A (RIDA) Activity and Cell Proliferation Rate: Role of Lysine Succinylation of RIDA

Luisa Siculella, Laura Giannotti, Benedetta Di Chiara Stanca, Matteo Calcagnile, Alessio Rochira, Eleonora Stanca, Pietro Alifano and Fabrizio Damiano

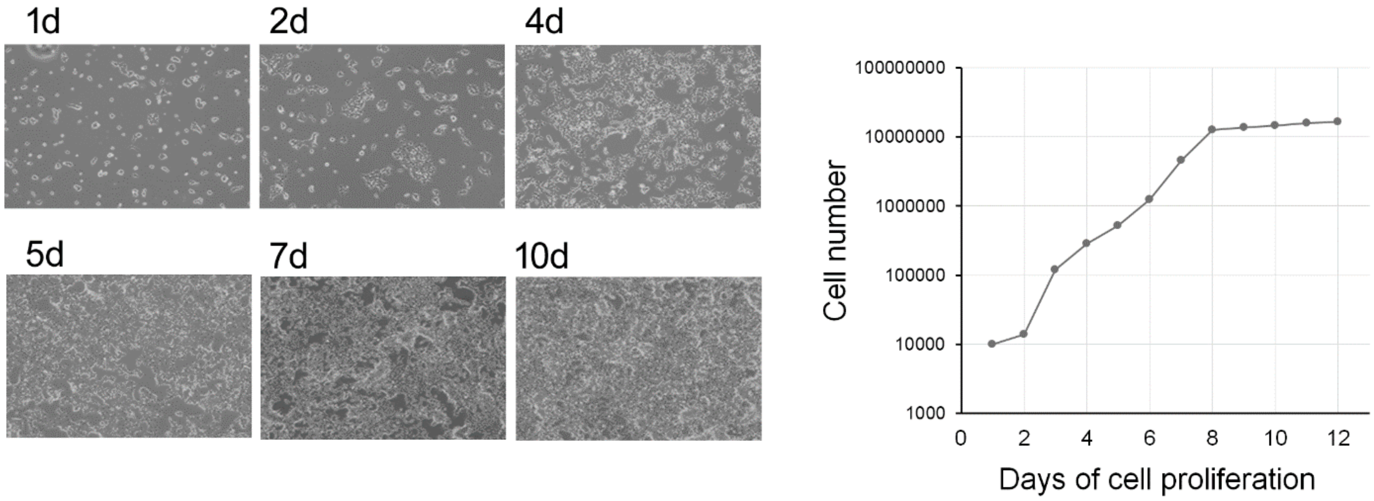

Figure S1. HEK cell proliferation curve. HEK293 proliferation curve was obtained by seeding and counting cells in 1–12 days period.

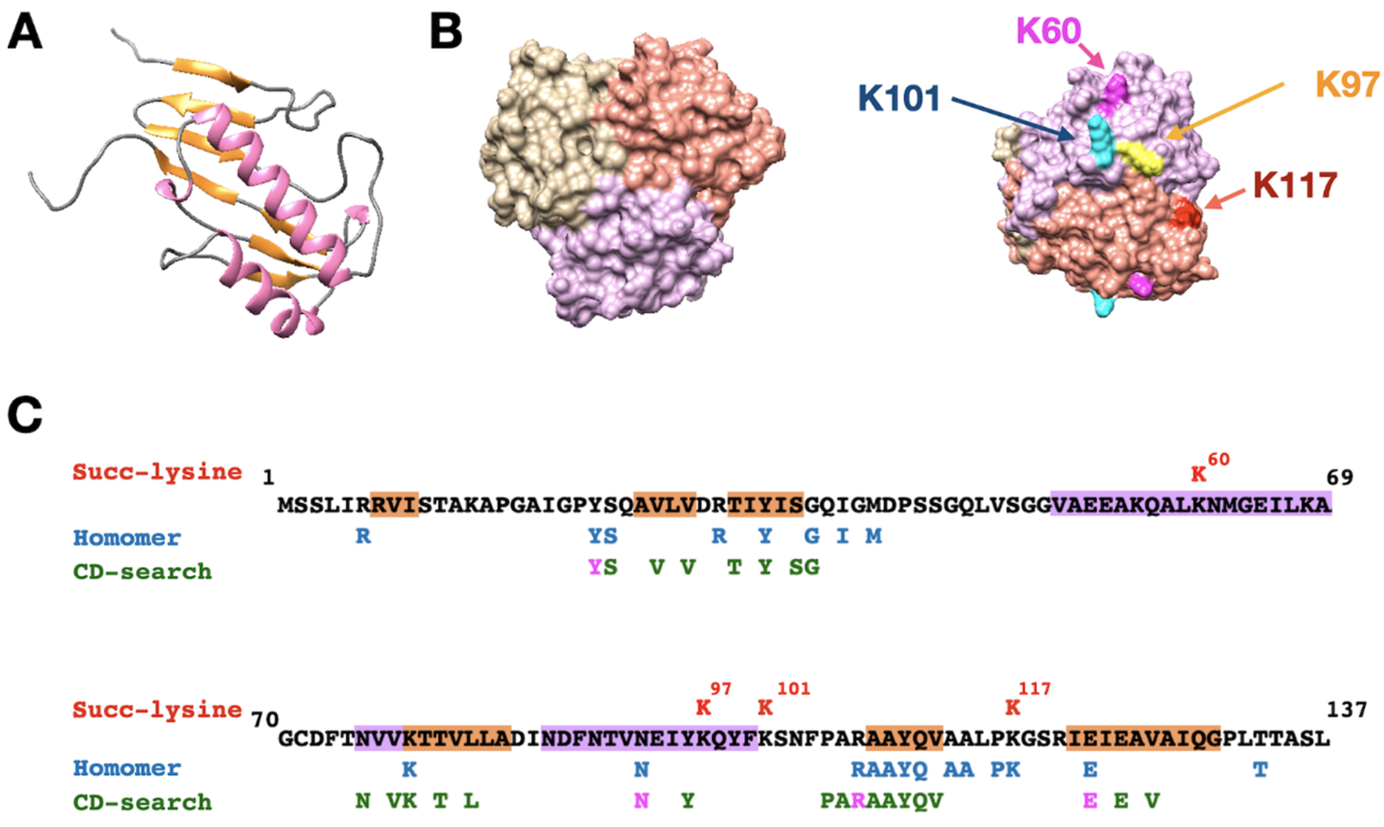

Figure S2. In silico analysis of hRIDA 3D trimeric structure. (**A**) The monomer structure of hRIDA, with the β-sheet (orange) and the α-helices (pink), is shown. (**B**) The predicted 3D model of unsuccinylated hRIDA trimer and the lysine residues involved in hRIDA succinylation are reported. (**C**) The amino acids at the interface of two contiguous monomers involved in trimer stabilization, predicted by Homomer (blue letters) and CD-search (green letters) programs, were reported. The predicted amino acids of active site were indicated in magenta. Amino acids in β-sheet and α-helices are depicted in orange and in purple boxes, respectively. hRIDA succinylated-lysine residues K60, K97, K101 and K117 are also indicated.

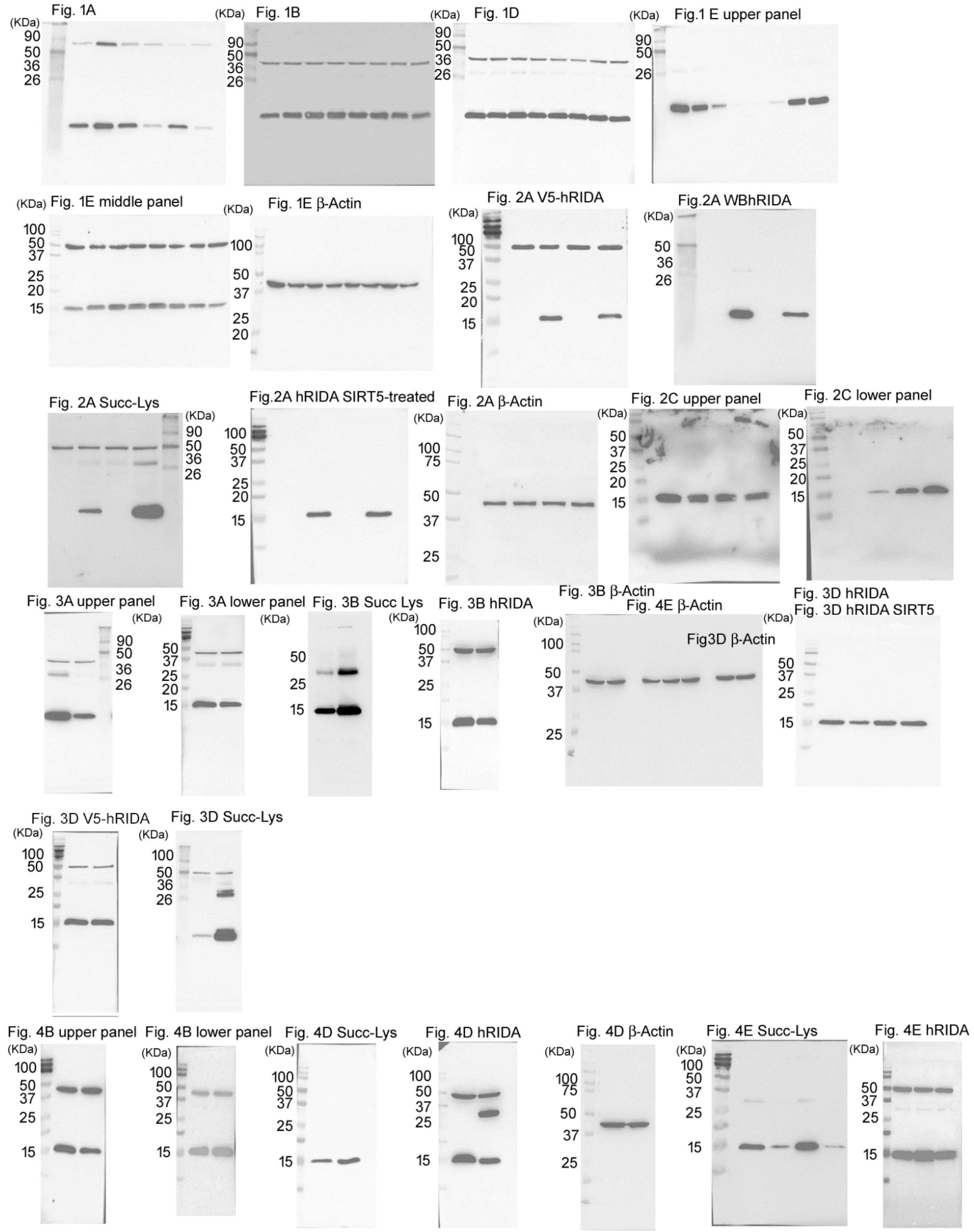

Figure S3. Western blots with marker sizes.
